# Achievement of sufficient antibody response after a fourth dose of wild‐type SARS‐CoV‐2 mRNA vaccine in nursing home residents

**DOI:** 10.1002/iid3.962

**Published:** 2023-08-28

**Authors:** Yong Chong, Takeyuki Goto, Haruka Watanabe, Naoki Tani, Akiko Yonekawa, Hideyuki Ikematsu, Nobuyuki Shimono, Yosuke Tanaka, Koichi Akashi

**Affiliations:** ^1^ Department of Medicine and Biosystemic Science Kyushu University Graduate School of Medical Sciences (The First Department of Internal Medicine) Fukuoka Japan; ^2^ Department of Clinical Immunology, Rheumatology, and Infectious Disease Kyushu University Hospital Fukuoka Japan; ^3^ Division of Influenza Research Japan Physicians Association Tokyo Japan; ^4^ Department of Center for the Study of Global Infection Center for the Study of Global Infection, Kyushu University Hospital Fukuoka Japan; ^5^ Department of Internal Medicine Medical Corporation SOUSEIKAI, Kanenokuma Hospital Fukuoka Japan

**Keywords:** antibody response, COVID‐19, fourth vaccination, nursing home residents, SARS‐CoV‐2, second booster

## Abstract

**Background:**

Infection control during COVID‐19 outbreaks in nursing facilities is a critical public health issue. Antibody responses before and after the fourth (second booster) dose of wild‐type severe acute respiratory syndrome coronavirus 2 (SARS‐CoV‐2) vaccine in nursing home residents have not been fully characterized.

**Methods:**

This study included 112 individuals: 54 nursing home residents (mean age: 84.4 years; 35 SARS‐CoV‐2‐naive and 19 previously infected) and 58 healthcare workers (mean age: 47.7 years; 25 SARS‐CoV‐2‐naive and 33 previously infected). Antispike and antinucleocapsid antibody responses to messenger RNA vaccination were evaluated using serum samples collected shortly and 5 months after the third dose, and shortly after the fourth dose.

**Results:**

The median immunoglobulin G (IgG) level in SARS‐CoV‐2‐naive residents was similar to that in SARS‐CoV‐2‐naive healthcare workers after the fourth dose (24,026.3 vs. 30,328.6 AU/mL, *p* = .79), whereas after the third dose the IgG level of SARS‐CoV‐2‐naive residents was approximately twofold lower than that in SARS‐CoV‐2‐naive healthcare workers. In residents with previous SARS‐CoV‐2 infection, timing of infection in relation to vaccination affected the kinetics of antibody responses. Residents infected after the third dose showed the highest IgG levels after the fourth dose among all groups (median: 64,328.8 AU/mL), in contrast to residents infected before initiating vaccination with antibody levels similar to those of SARS‐CoV‐2‐naive residents.

**Conclusions:**

Advanced aged nursing home residents, poor responders in the initial SARS‐CoV‐2 vaccine series, could achieve sufficient antibody responses after the fourth (second booster) vaccination, comparable to those of younger adults.

## Graphical abstract

Antibody responses before and after the fourth (second booster) dose of wild‐type SARS‐CoV‐2 mRNA vaccine in nursing home residents. The median IgG level after the fourth dose in SARS‐CoV‐2‐naive residents was similar to that in SARS‐CoV‐2‐naive healthcare workers. The IgG ratio of SARS‐CoV‐2‐naive residents to healthcare workers increased from 0.5 (50%) after the third dose to 0.8 (80%) after the fourth dose.

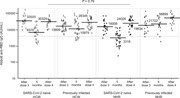

## 1 INTRODUCTION

The coronavirus disease (COVID‐19) pandemic, caused by severe acute respiratory syndrome coronavirus 2 (SARS‐CoV‐2) has affected long‐term care facility residents due to their advanced age, frailty, and co‐morbidities. Although a third (booster) dose of SARS‐CoV‐2 vaccines showed the efficient recovery of specific antibody responses and clinical efficacy in general population and nursing home residents,^1^, ^2^, ^3^ relatively rapidly diminishing efficacy after booster was reported.^4^ In such a situation, a fourth (second booster) dose of wild‐type SARS‐CoV‐2 vaccines has been administered in several countries. In Japan, the fourth vaccination was conducted for adults aged over 60 years and healthcare workers, mainly from June to August, 2022. The recovery of both postsecond booster antibody responses and vaccine efficacy in the general population has been shown.^5^, ^6^ However, the detailed kinetics of antibody levels after the third and fourth doses in nursing home residents have been little characterized.

Our previous study at a nursing facility in Japan where a COVID‐19 outbreak was experienced in the spring of 2020 showed that the specific antibody responses after the initial BNT162b2 messenger RNA (mRNA) vaccination in SARS‐CoV‐2‐naive nursing home residents were markedly lower than those in healthcare workers.^7^ In contrast, previously infected residents obtained rapid and robust antibody responses comparable to those of healthcare workers.^7^ Marked increases in antibody levels after the third vaccination in nursing home residents have also been reported.^8^ Here, we evaluated the antibody responses shortly after the third dose, 5 months after the third dose, and shortly after the fourth dose in the same cohort, including SARS‐CoV‐2‐naive and previously infected residents and healthcare workers. The facility suffered a second COVID‐19 outbreak during the epidemic period of omicron variants after the third dose. In this study, individuals infected during the second outbreak were newly added as previously infected members of the cohort. Detailed information on the immunogenicity of a series of SARS‐CoV‐2 vaccinations in nursing home residents could be useful for maintaining effective control measures for future COVID‐19 outbreaks in nursing homes.

## 2 METHODS

### 2.1 Participants and sample collection

This study was conducted as a serological follow‐up evaluation after reporting specific antibody responses after the initial two‐dose SARS‐CoV‐2 vaccine series and third dose at a nursing home with an outbreak in April, 2020.^9^ Individuals of this cohort, whose samples were obtained 5 months after the third dose and shortly after the fourth dose were included in this follow‐up evaluation. Some nursing home residents and healthcare workers in the facility were infected with SARS‐CoV‐2 during the second outbreak that occurred in early to mid‐April, 2022. Previously infected nursing home residents and healthcare workers in this study included individuals infected during the first outbreak in April, 2020, before receiving the initial vaccine dose and during the second outbreak in April, 2022, after receiving the third dose. The diagnosis of infection in previously infected individuals are shown in a previous report.^7^ The initial BNT162b2 (Pfizer‐BioNTech) mRNA vaccination was performed twice at a 21‐day interval from May to July, 2021. The third (booster) vaccination program was conducted from January to February, 2022 using the same BNT162b2 vaccine. The fourth (second booster) vaccination was performed from July to August, 2022, approximately 6 months after the third dose, using either the BNT162b2 or mRNA‐1273 (Moderna) vaccine. Collection of serum samples was scheduled on day 21 after the fourth dose. Clinical data, including information on underlying diseases (Table 1), were collected from medical records and questionnaires. Serological testing and accompanying clinical data collection were based on the consent obtained from participants or family members and approval by the Ethics Committee of the Hara‐doi Hospital.

**Table 1:** Demographic characteristics in healthcare workers and nursing home residents.

	SARS‐CoV‐2‐naive	Previously infected	SARS‐CoV‐2‐naive	Previously infected
Characteristic	HCW	HCW	NHR	NHR
Total number	25	33	35	19
Gender, male	13 (52.0)	11 (33.3)	10 (28.6)	8 (42.1)
Age, mean years ± *SD* (range)	48.7 ± 13.6 (27–70)	47.0 ± 11.0 (21–65)	84.5 ± 6.6 (68–94)	84.2 ± 9.9 (62–100)
Chronic underlying disease				
No disease	22 (88.0)	28 (84.9)	0 (0.0)	0 (0.0)
Dementia	0 (0.0)	0 (0.0)	35 (100.0)	18 (94.7)
HDS‐R (0–30)^a^, mean scores	N/A	N/A	4.0	6.0
Hypertension	2 (8.0)	3 (9.1)	9 (25.7)	5 (26.3)
Cardiac disease	1 (4.0)	0 (0.0)	5 (14.3)	2 (10.5)
Chronic pulmonary disease	0 (0.0)	2 (6.1)	4 (11.4)	1 (5.3)
Renal disease	0 (0.0)	0 (0.0)	3 (8.6)	0 (13.3)
Cerebrovascular disease	0 (0.0)	0 (0.0)	5 (14.3)	6 (31.6)
Diabetes	0 (0.0)	2 (6.1)	4 (11.4)	3 (15.8)
Time from vaccination to sample collection,				
mean days ± *SD* (range)				
Scheduled 21 days after the third dose	21.1 ± 1.24 (19–23)	20.8 ± 1.16 (18–24)	20.4 ± 0.50 (20–21)	20.4 ± 1.42 (15–21)
Scheduled 5 months after the third dose	155.6 ± 4.37 (149–168)	158.9 ± 3.72 (154–169)	149.1 ± 6.69 (140–166)	150.3 ± 7.07 (143–165)
Scheduled 21 days after the fourth dose	19.9 ± 0.46 (19–21)	20.5 ± 1.89 (18–25)	19.2 ± 0.83 (19–21)	20.1 ± 0.72 (19–21)
Time between the third and fourth doses,	189.8 ± 23.53 (157–221)	202.0 ± 16.09 (161–225)	168.6 ± 8.17 (163–189)	173.0 ± 9.2 (151–187)
mean days ± *SD* (range)				

*Note*: Data are no. (%) of individuals, unless indicated otherwise. Dementia was defined as a score of HDS‐R ≤ 20 (total maximum score of 30).

Abbreviations: HCW, healthcare worker; HDS‐R, the revised Hasegawa's dementia scale; N/A, not applicable; NHR, nursing home resident; SARS‐CoV‐2, severe acute respiratory syndrome coronavirus 2.

^a^ HDS‐R is a screening test for age‐associated dementia.

### 2.2 Serological testing

Serological testing was performed using the collected serum samples. The quantitative levels of immunoglobulin G (IgG) antibodies for the spike (receptor‐binding domain) and nucleocapsid antigens of SARS‐CoV‐2 were examined using the Abbott Architect immunoassays (SARS‐CoV‐2 IgG II Quant and SARS‐CoV‐2 IgG, Abbott). When the assay range was >40,000 AU/mL, the samples tested were diluted and re‐measured. The antispike IgG levels of ≥4160 AU/mL were used as a surrogate marker of highly effective antibody neutralization, based on the manufacturer's instruction. This threshold corresponded to a 0.95 probability of obtaining a plaque reduction neutralization test (PRNT) ID_50_ at a 1:250 dilution. Antinucleocapsid antibody testing for samples from SARS‐CoV‐2‐naive individuals was used as a reference for recategorizing them as previously infected, as well as for antispike antibody testing.

### 2.3 Statistical analysis

Categorical variables were analyzed using Fisher's exact test. Continuous variables were compared using the Wilcoxon rank‐sum test. All *p* values of <.05 were considered statistically significant. All statistical analyses were performed using JMP Pro, version 14 (SAS Institute, Inc.).

## 3 RESULTS

A total of 112 individuals, including 58 healthcare workers (mean age: 47.7 years; 25 SARS‐CoV‐2‐naive and 33 previously infected) and 54 nursing home residents (mean age: 84.4 years; 35 SARS‐CoV‐2‐naive and 19 previously infected) were eligible for the study. Based on antispike and antinucleocapsid antibody testing, none of the SARS‐CoV‐2‐naive healthcare workers or residents were recategorized as previously infected. The baseline clinical characteristics of the 112 individuals are shown in Table 1. No individuals received immunosuppressant treatment, except for a previously infected healthcare worker with steroid treatment. All durations from vaccination to sample collection were similar among the healthcare workers and residents. The periods between the third and fourth doses were also similar, at approximately 6 months.

**Figure 1:**
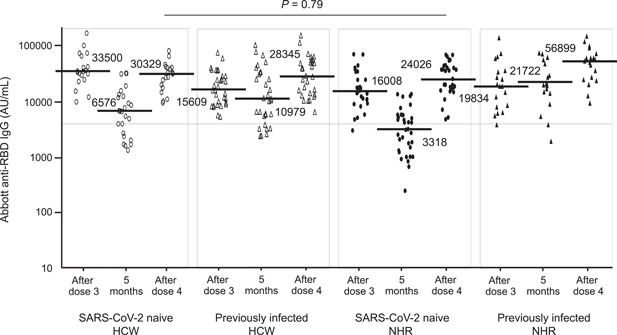
Antispike (RBD) IgG antibody levels shortly after the third dose, 5 months after the third dose, and shortly after the fourth dose of wild‐type SARS‐CoV‐2 mRNA vaccination in SARS‐CoV‐2‐naive and previously infected healthcare workers and nursing home residents. The white circles indicate data of SARS‐CoV‐2‐naive healthcare workers (*n* = 25). The white triangles indicate data of previously infected healthcare workers (*n* = 33). The black circles indicate data of SARS‐CoV‐2‐naive residents (*n* = 35). The black triangles indicate data of previously infected residents (*n* = 19). After doses 3 and 4 indicate 21 days after each dose. Five months indicate 5 months after the third dose. The horizontal solid bars and numbers in each group indicate the median values. The horizontal line indicates the value of 4160 AU/mL, a threshold level indicating highly effective antibody neutralization. HCW, healthcare worker; IgG, immunoglobulin G; mRNA, messenger RNA; NHR, nursing home resident; RBD, receptor‐binding domain; SARS‐CoV‐2, severe acute respiratory syndrome coronavirus 2

Figure 1 shows the antispike IgG antibody levels shortly and 5 months after the third dose and shortly after the fourth dose. The median IgG level in SARS‐CoV‐2‐naive residents 5 months after the third dose was approximately twofold lower than that in SARS‐CoV‐2‐naive healthcare workers, with the lowest levels among the four groups. However, the frequency of IgG levels of ≥4160 AU/mL in the residents 5 months after the third dose was almost 50% (48.6%, 17/35), in contrast to 6 months after the second dose.^8^ The threshold of antispike IgG levels of ≥4160 AU/mL corresponded to a 0.95 probability of obtaining a PRNT ID_50_ at a 1:250 dilution. The median IgG level after the fourth dose in SARS‐CoV‐2‐naive residents appeared to be lower than that in SARS‐CoV‐2‐naive healthcare workers, but this difference was not statistically significant (24,026.3 vs. 30,328.6 AU/mL, *p* = .79). As a result, the IgG levels after the fourth dose in SARS‐CoV‐2‐naive residents reached a comparable level of healthcare workers, from approximately half the level after the third dose. IgG levels in previously infected residents seemed not to decrease at a time of 5 months after the third dose, in contrast to SARS‐CoV‐2‐naive residents. The median IgG level in previously infected residents after the fourth dose was the highest among the four groups. Basic data on the antibody levels are shown in Table S1.

**Figure 2:**
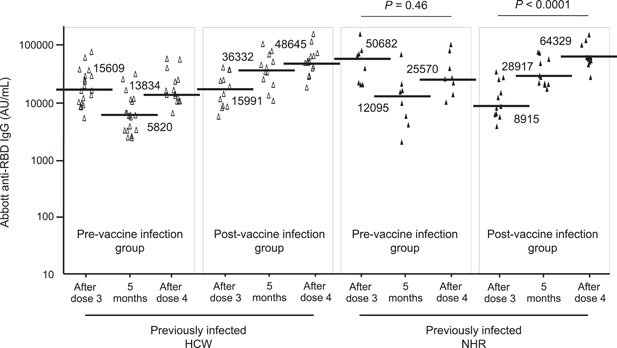
Antispike (RBD) IgG antibody levels shortly after the third dose, 5 months after the third dose, and shortly after the fourth dose of wild‐type SARS‐CoV‐2 mRNA vaccination in previously infected healthcare workers and nursing home residents. Previously infected healthcare workers consist of a group infected before the initial vaccine dose (prevaccine infection group, *n* = 19) and a group infected after the third dose (postvaccine infection group, *n* = 14). Previously infected nursing home residents consist of a group infected before the initial vaccine dose (prevaccine infection group, *n* = 8) and a group infected after the third dose (postvaccine infection group, *n* = 11). After doses 3 and 4 indicate 21 days after each dose. Five months indicate 5 months after the third dose. The horizontal solid bars and numbers in each group indicate the median values. HCW, healthcare worker; IgG, immunoglobulin G; mRNA, messenger RNA; NHR, nursing home resident; RBD, receptor‐binding domain; SARS‐CoV‐2, severe acute respiratory syndrome coronavirus 2

Previously infected healthcare workers and nursing home residents were divided into two groups based on the time of infection: a group infected before the initial vaccine dose (prevaccine infection group) and a group infected after the third dose (postvaccine infection group), and their IgG levels were compared (Figure 2). The median IgG level of residents in the prevaccine infection group decreased by approximately 80% at a time of 5 months after the third dose, similar to that of SARS‐CoV‐2‐naive residents. IgG levels directly after the third and fourth doses were comparable in the prevaccine infection group (*p* = .46), although a definite increase before (5 months after the third dose) and after the fourth dose was found. Healthcare workers in the prevaccine infection group showed a tendency similar to that of the same group residents. In contrast, IgG levels of residents in the postvaccine infection group significantly increased 5 months after the third dose, compared to just after the third dose (28,916.6 vs. 8914.9 AU/mL in a median level, *p* = .004). Additionally, a further increase in IgG levels before and after the fourth dose was observed in postvaccine infection group residents, with the highest level after the fourth dose among all groups, including SARS‐CoV‐2‐naive and previously infected individuals (64,328.8 vs. 28,916.6 AU/mL in a median level, *p* = .017). Healthcare workers in the postvaccine infection group showed a tendency similar to that of the same group residents.

## 4 DISCUSSION

In SARS‐CoV‐2‐naive nursing home residents in our cohort, the rate of decrease in antispike antibody levels over time after the third (booster) vaccination was similar to that after the second dose (a decrease of approximately 90% 6 months after each dose).^8^ However, the frequency of antibody levels ≥4160 AU/mL in the residents after the third dose was much higher than after the second dose, even 6 months after vaccination, because the antibody levels increased approximately five times after the third dose compared with the levels after the second dose.^8^ This finding might have contributed to protection against infection. The definite antibody response induced by the fourth (second booster) vaccination in SARS‐CoV‐2‐naive residents is noteworthy. Our previous study showed a robust increase in antibody levels after the third dose in these residents.^8^ These findings provide evidence that the ability to induce a sufficient antibody response persists in SARS‐CoV‐2‐naive residents, even after the fourth dose. This implies that vaccine‐derived SARS‐CoV‐2‐specific memory function remains and elicits an adequate response at the time of 6 months after the third vaccination. In a series of cohort studies, the IgG ratio of SARS‐CoV‐2‐naive residents to healthcare workers after the second, third, and fourth doses became one‐fifth, one‐half, and comparable, respectively.^7^, ^8^ These results suggest that in advanced aged nursing home residents, antibody productivity induced by vaccination against SARS‐CoV‐2 is not completely reduced. In Japan, omicron variants, BA.1 and BA.5, were prevalent in 2022, at the time that the third and fourth doses, respectively, were administered. Circulating omicron variants, including BA.1 and BA.5, have been shown to significantly change the antigenicity compared with the wild‐type strain.^10^, ^11^ The third and fourth vaccine doses have been shown to definitely increase neutralizing activities against BA.1 and BA.5, as well as the wild type.^10^, ^11^ An increase in specific antibody responses after the fourth SARS‐CoV‐2 vaccination in nursing home residents could be beneficial for protection against omicron variant infection.

Our previous study found that antibody levels comparable to healthcare workers were induced by the initial vaccine series in previously infected residents (April, 2020 before vaccination), in contrast to SARS‐CoV‐2‐naive residents.^7^ In this study, the antibody levels after the fourth dose in residents infected before the initial vaccination (prevaccine infection group) were similar to those in SARS‐CoV‐2‐naive residents. This finding indicates that in previously infected residents with preantigen exposure, the advantage of obtaining rapid and robust antibody responses after the first dose had been lost by the time of the fourth dose. We cannot accurately elucidate the underlying mechanism, requiring a further investigation to address this issue. Notably, the result of antibody levels before and after the fourth dose in previously infected residents in the postvaccine infection group (April, 2022 after the third vaccination) were contrasting compared with that in the prevaccine infection group. IgG levels in the postvaccine infection group residents at the time of 5 months after the third dose were comparable to those in SARS‐CoV‐2‐naive residents after the fourth dose. This increase in antibody levels could be attributed to omicron variant infection. Thus, the infection with omicron variants, in which the antigenicity of the spike protein differs from that of the wild‐type, appears to have induced wild‐type‐derived antispike antibody based on vaccine immunity. This possibility is supported by results from other studies.^12^ The acquisition of “hybrid‐immunity” due to SARS‐CoV‐2 infection after vaccination may elicit a robust immune response. Further studies are required to elucidate the underlying mechanism whereby different timing of infection in relation to vaccination elicits different immune responses.

A limitation of our study is the small sample size. We could not examine neutralizing activities against omicron variants for antispike antibody levels elicited by the fourth dose of wild‐type SARS‐CoV‐2 vaccine. In addition, there has been a bias of survivorship of nursing home residents that are able to survive prior COVID‐19 and/or those that also survive over time to receive the subsequent booster doses. Based on these limitations, the conclusions and generalizability of this study may be limited. The fourth vaccination, which was conducted during an omicron‐predominant period (BA.1 and BA.5), is likely to have provided a meaningful effect on protecting nursing home residents from infection, considering reports on a definite increase in neutralizing activities against omicron variants and a recovery of vaccine efficacy for preventing omicron variant infection.^11^, ^13^ Current epidemic omicron variants, mainly BQ.1.1 and XBB.1, have further changed their antigenicity, with a remaining concern regarding the recovery of neutralizing activities after booster vaccination. Omicron‐containing bivalent booster vaccination has been conducted in several countries, including Japan. We plan to evaluate the kinetics of antibody levels before and after the bivalent vaccine dose.

## AUTHOR CONTRIBUTIONS

**Yong Chong**: Conceptualization (lead); data curation (equal); formal analysis (lead); investigation (equal); methodology (equal); Writing‐original draft (lead). **Takeyuki Goto**: Investigation (equal); methodology (equal). **Haruka Watanabe:** Investigation (equal); methodology (equal). **Naoki Tani**: Investigation (equal); methodology (equal). **Akiko Yonekawa**: Data curation (equal). **Hideyuki Ikematsu**: Conceptualization (supporting); supervision (equal); validation (equal); Writing‐review and editing (equal). **Nobuyuki Shimono**: Supervision (equal); validation (equal); Writing‐review and editing (equal). **Yosuke Tanaka**: Conceptualization (supporting); supervision (equal); validation (equal). **Koichi Akashi**: Project administration (lead).

## CONFLICT OF INTEREST STATEMENT

The authors declare no conflicts of interest.

## Supporting information

Supporting information.

## Data Availability

Data will be made available on request.
